# Exploring Virtual Reality and Exercise Simulator Interventions in Patients With Attention Deficit Hyperactivity Disorder: Comprehensive Literature Review

**DOI:** 10.2196/57297

**Published:** 2025-01-29

**Authors:** Gurdeep Sarai, Prem Prakash Jayaraman, Oren Tirosh, Nilmini Wickramasinghe

**Affiliations:** 1 School of Health Sciences Swinburne University of Technology Melbourne Australia; 2 School of Science, Computing and Engineering Technologies Factory of the Future and Digital Innovation Lab Swinburne University of Technology Melbourne Australia; 3 Department of Health and Biomedical Sciences Clinical Biomechanics and Rehabilitation RMIT University Melbourne Australia; 4 School of Computing, Engineering and Mathematical Sciences Optus Chair Digital Health La Trobe University Melbourne Australia

**Keywords:** exercise-based simulator, exergame, virtual reality, physical activity, attention-deficit/hyperactivity disorder

## Abstract

**Background:**

This review explores virtual reality (VR) and exercise simulator–based interventions for individuals with attention-deficit/hyperactivity disorder (ADHD). Past research indicates that both VR and simulator-based interventions enhance cognitive functions, such as executive function and memory, though their impacts on attention vary.

**Objective:**

This study aimed to contribute to the ongoing scientific discourse on integrating technology-driven interventions into the management and evaluation of ADHD. It specifically seeks to consolidate findings on how VR and exercise simulators may support individuals with ADHD, acknowledging associated challenges and implications inherent in both technological approaches.

**Methods:**

This research looks at existing literature to examine the potential efficacy of VR and exercise simulator–based interventions for individuals with ADHD. It evaluates the capacity of these interventions to address specific challenges along with an emphasis on the adjustments for accommodating unique user behaviors. Additionally, it underscores the limited exploration of user perceptions of exercise simulator–based interventions and the undervalued role of motor function in both ADHD assessment and symptom management.

**Results:**

The findings of this scoping review reveal that, while these interventions enhance user motivation and enjoyment, certain challenges resist modification through technology. Furthermore, this study explores the intricate complexities involved in customizing these technologies to accommodate the diverse aspects of user behavior and highlights the potential limitations in the use of VR.

**Conclusions:**

This scoping review contributes to the ongoing research on enhancing interventions to support individuals with ADHD. It advocates for participant-centric approaches that aim to optimize both cognitive and motor outcomes while prioritizing the enhancement of user experiences. This study emphasizes the need for a comprehensive approach to interventions, recognizing the relationship between cognitive and motor abilities, and calls for improving technological interventions to address the varied needs of individuals with ADHD.

## Introduction

Attention-deficit/hyperactivity disorder (ADHD) continues to be a prevalent neurodevelopmental condition, marked by persistent patterns of inattention or hyperactivity-impulsivity that interfere with an individual’s functioning and development [1]. Traditional approaches toward managing ADHD involve a combination of therapeutic strategies and medication. However, achieving sustained patient focus and commitment to these treatments can be challenging. Physical activity and exercise have been acknowledged as beneficial nonpharmacological adjuncts to traditional ADHD therapies, contributing to enhancing cognitive function, behavior, and physical well-being [2]. However, it can be especially difficult for people with ADHD to maintain consistent and interesting physical activity due to the symptoms they face, such as impulsivity, short attention span, and hyperactivity. Thus, there is a growing demand for new and captivating interventions. In various health care sectors, inventive strategies involving the use of exercise-based simulators and virtual reality (VR) are being progressively examined and employed [3]. These technological advancements provide engaging and immersive environments, which may raise adherence and active participation in physical activity.

In addition, it is noteworthy that the timing deficits and motor skill impairments commonly associated with ADHD could be addressed through these interactive interventions. Noreika et al [4] provided evidence suggesting that dysfunctions in the neural networks responsible for timing tasks are integral to ADHD, affecting a wide range of cognitive operations, including motor timing, time perception, and temporal foresight. Therefore, therapeutic strategies incorporating physical activities facilitated by simulators and VR could not only capture attention but also help improve motor proficiency and timing deficits. By promoting movement coordination and rhythm, these technologies might assist in reinforcing the neural circuits that underpin time processing and motor skills, presenting a complementary approach to traditional ADHD interventions.

Physical exercise is a key factor for children with ADHD, playing a crucial role in developing brain structure and function, which in turn influences the developmental trajectory of the disorder [5]. Regular participation in physical activity improves the flow of oxygen to the brain and enhances connections between neurons, particularly supporting development of the frontal lobe [5,6]. This area is important for cognitive processing and is often underdeveloped in individuals with ADHD. Exercise may improve executive functions like inhibitory control, working memory, and cognitive flexibility, by increasing the essential neurotransmitters dopamine and norepinephrine, which are pivotal for attention and concentration [7]. Moreover, physical activity enhances the processing of external information, improves coordination, and can reduce impulsivity and aggressive behaviors by establishing structured behavioral patterns [8]. Without these benefits, children with ADHD risk continued struggles with attention, learning difficulties, motor skills deficits, and disruptive behavior, which can seriously inhibit their cognitive and social development [5]. Compounding the importance of exercise, the systematic review conducted by Cortese et al [9] identified a noteworthy link between obesity and ADHD, suggesting shared underlying factors, including dopaminergic dysregulation and oxidative stress, and underscoring the necessity of addressing obesity risk in ADHD management and vice versa. This association highlights the urgency for continued research into the complex interrelations among ADHD, exercise, obesity, and the potential long-term effects of ADHD medications on weight, advocating for an integrated and multifaceted approach to treatment [9]. In parallel with this perspective, VR and exercise-based simulators have emerged as progressive tools in various fields, including fitness, health, and rehabilitation, offering new avenues for such an approach in the management of ADHD.

VR and exercise-based simulators have grown in popularity over the last few years, especially in various fields of fitness, health, and rehabilitation. These innovative technologies offer interactive and immersive environments that provide users with multisensory feedback and real-time performance tracking. The main purpose of these systems is to enhance user experience while optimizing the effectiveness of physical training interventions [10]. While both VR and exercise-based simulators aim to improve physical training outcomes, they do differ in certain aspects. For instance, VR typically involves the use of a VR headset that immerses users in computer-generated environments [11]. Exercise-based simulators are advanced physical devices designed to imitate and recreate the movements and activities associated with exercise routines or sports [12]. These sophisticated simulators aim to replicate the complex motion, resistance, and environment of real-world exercises to enhance skill acquisition and promote physical development. By closely mimicking the intricacies of actual exercise movements, these simulators provide users with a realistic training experience that can improve their overall fitness level [12]. Furthermore, these devices offer opportunities for individuals to engage in targeted practice sessions that focus on specific skills or aspects of physical performance. The immersive nature of exercise simulators creates an optimal setting for individuals to develop proficiency while also enjoying a virtual yet highly tangible workout experience [13]. Beyond their physical benefits, these simulators are increasingly harnessed for cognitive training, offering a diverse range of exercises that are designed to stimulate mental processes, enhance neuroplasticity, and contribute to cognitive rehabilitation.

However, it is only through this extensive scoping review that we can begin to uncover the potential effects, applications, limitations, and future opportunities associated with these cutting-edge technologies. Specifically, we have applied the PICO (Population, Intervention, Comparison, Outcome) framework to structure our research question and methodology [14]. We aim to address the following research question: In individuals with ADHD (Population), can immersive forms of technology (Intervention), such as VR and exercise-based simulators (Comparison), lead to improvements in cognitive and motor function (Outcome)? This comprehensive analysis of the literature will explore the effectiveness, benefits, and potential drawbacks of these interventions, ultimately informing future research and clinical practice.

## Methods

### Design

We performed a scoping review following the methodology outlined in the PRISMA-ScR (Preferred Reporting Items for Systematic Reviews and Meta-Analyses Extension for Scoping Reviews) guidelines [15]. The PRISMA-ScR checklist is provided in Multimedia Appendix 1. The PRISMA-ScR statement includes a 27-item checklist and a 4-phase flow diagram to ensure transparency and complete reporting in systematic reviews. Our protocol was drafted in accordance with these guidelines and was revised by the research team. The final protocol was registered prospectively with the Open Science Framework on July 19, 2024 [16].

### Eligibility Criteria

To be included in the review, papers needed to focus on the use of VR or exercise-based simulators for managing the symptoms of ADHD. Peer-reviewed journal papers were included if they met the following criteria: published between 2019 and 2023; written in English; involved human participants diagnosed with ADHD; and described an intervention using VR, a motion-based device, or exercise-based simulators. Studies were included if they reported on outcomes related to cognitive and motor functions, such as attention, inhibitory control, cognitive flexibility, processing speed, motor skills, and physical fitness. Quantitative, qualitative, and mixed-method studies were considered to capture various aspects of the effectiveness of the interventions. Papers were excluded if they did not specifically focus on ADHD; involved nonhuman participants; or described interventions that did not use VR, a motion-based device, or exercise-based simulators. Studies focusing on other neurodevelopmental disorders or specific learning disabilities were also excluded unless they included a distinct analysis for ADHD participants.

### Information Sources

To identify potentially relevant documents, the following bibliographic databases were searched from 2019 to April 2023: PubMed, Scopus, PsycINFO, and Web of Science. The search strategies were drafted and further refined through team discussions. The final search results were exported into EndNote, and duplicates were removed. The electronic database search was supplemented by scanning relevant reviews and reference lists of included studies to identify additional sources.

### Literature Search Strategy

The literature search was conducted across the following major electronic databases: PubMed, Scopus, PsycINFO, and Web of Science. The search covered years from 2020 up until July 2023. The search strategy employed in our review consistently followed a structured approach. The base search string included the population terms “ADHD” and “attention-deficit/hyperactivity disorder,” combined with intervention-related keywords such as “virtual reality,” “VR,” “exercise simulator,” “exergaming,” and “serious games.” To refine the focus on relevant outcomes, additional terms were incorporated, including “cognitive function,” “motor skills,” “executive function,” and “physical activity.” This search string was systematically applied across all databases to ensure comprehensive retrieval of studies addressing the intersection of ADHD, VR, and exercise simulator interventions. We used Boolean operators such as “AND” to combine different concepts (eg, exercise simulators AND VR) and “OR” to include alternative terms (eg, ADHD OR attention-deficit/hyperactivity disorder). Articles were included if they focused on ADHD interventions using VR or exercise simulators. Studies were excluded if they were not in English, focused on non-ADHD populations, or were review articles without original data. This comprehensive search strategy ensured a thorough examination of relevant literature.

### Selection Process

To ensure a rigorous and standardized selection process for the included sources of evidence, we developed a detailed selection spreadsheet. This included specific questions to evaluate the relevance and quality of the sources, such as the focus on VR or exercise-based simulators for ADHD, type of study, reported outcomes, and keywords. The spreadsheet was initially used by 1 reviewer on a random sample of 979 full-text articles. The results were then discussed with another reviewer, and the form was refined to resolve any inconsistencies and to ensure clarity. Key changes included clarifying the criteria for inclusion and exclusion and adding more specific questions related to the interventions and outcomes.

To initiate the full screening process, duplicate articles were first eliminated from consideration. Subsequently, titles and then abstracts of potential articles underwent a thorough assessment to exclude any irrelevant publications. Publications were then once again crosschecked with our inclusion and exclusion criteria. Any disagreements that arose during the screening process were resolved through collaborative decision-making. If the reviewers were unable to reach a consensus, a third reviewer was brought in to help determine the appropriate course of action. This approach ensured that all included studies met the predefined eligibility criteria. Microsoft Excel was used for managing and charting records during the data charting process, while the software EndNote was used for managing selected references and tracking records after the eligibility stage of the flowchart.

### Data Charting and Items

A data charting form was jointly developed to determine which variables to extract for the review. The initial form included categories such as “search typed in/keyword, title, database/search engine/top-level source, platform/publisher, date, range of coverage from source, link, methodology, strengths, weaknesses, data sets, data analysis, sample size, target population and area, theory, and personal interest.” This comprehensive set of categories was designed to capture all relevant aspects of each source and to ensure consistency and thoroughness in data extraction.

The data charting was conducted using Microsoft Excel, which allowed for efficient organization and manipulation of the extracted data. Calibration was carried out by having 2 supervisors review and provide feedback on the initial charting form. This feedback was used to refine the form iteratively. Each reviewer independently charted data from a sample set of sources to identify any discrepancies or areas for improvement in the form. The results of these independent charting exercises were discussed in detail, and the form was continuously updated to address any inconsistencies and to ensure clarity and comprehensiveness.

Three individuals were involved in the charting process: GS served as the primary charting agent, while 2 supervisors reviewed the charted data. The primary charting was done independently, and the supervisors provided periodic reviews to ensure accuracy and consistency. Any inconsistencies identified during these reviews were resolved through discussion and consensus, ensuring that all data entries were accurate and aligned with the review’s objectives.

Throughout the process, any items that required interpretation, such as subjective assessments of strengths and weaknesses or methodological nuances, were carefully documented. These interpretative items were discussed among the team to reach a consensus, ensuring that the final data entries were as objective and consistent as possible.

Revisions to the charting form were made iteratively based on the feedback and findings during the initial calibration exercises. For instance, some categories were refined to provide clearer definitions or to better capture the nuances of the data. The rationale for these changes was to enhance the reliability and validity of the data extraction process.

### Data Synthesis

We synthesized the range of evidence included in this review to answer our research questions and objectives by grouping the studies based on their intervention type: VR, motion-based devices, and exercise-based simulators. For each group, we summarized the study characteristics in terms of sample, focus of the study, trials and duration, and outcomes. This structured approach allowed us to systematically compare the various interventions and their effectiveness.

The evidence from the included studies has been presented in tables for clarity and ease of comparison. The tables include columns for sample, focus of the study, trials and duration, and outcomes, allowing for a quick visual representation of the key aspects of each intervention type. This approach facilitates a comprehensive understanding of the strengths, weaknesses, and overall effectiveness of the different interventions.

## Results

### Included Studies

**Figure 1:**
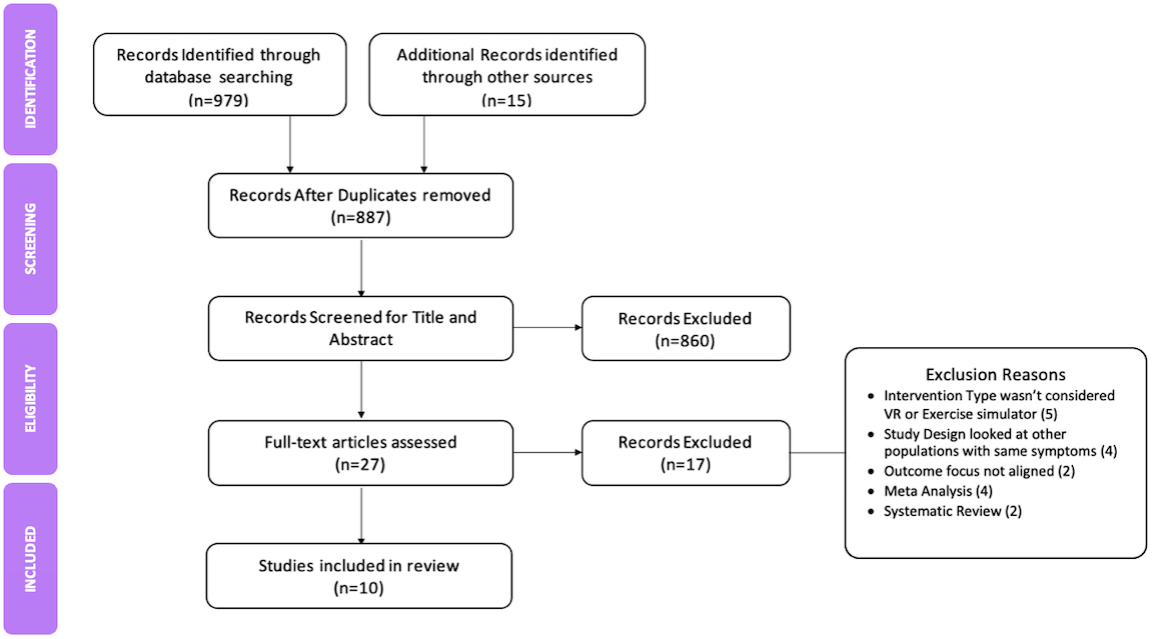
PRISMA (Preferred Reporting Items for Systematic Reviews and Meta-Analyses) flow diagram.

Out of the initial 994 articles that were identified, a total of 10 were selected and re-evaluated for eligibility (Figure 1). The research designs used in these studies included randomized controlled trial, comparative study, pilot study, and exploratory research study. Throughout this process, a recurring emphasis on cognitive function and attention, as well as some aspects of motor function, in relation to the use of both simulators and VR, became apparent.

### VR Interventions for ADHD

A summary of VR interventions for ADHD is presented in Table 1. The study by Rodrigo-Yanguas et al [17] used “The Secret Trail of Moon,” a VR game specifically designed for ADHD treatment, comprising various mini-games aimed at enhancing cognitive functions such as inhibitory control, selective attention, cognitive flexibility, and processing speed. Participants reported high levels of enjoyment, suggesting the potential of VR games as engaging therapeutic tools for ADHD management. Similarly, Bernardelli et al [18] assessed a VR-based serious game designed to enhance attention and cognitive skills in children with ADHD, confirming the high feasibility and usability of the application. This study indicates potential future improvements in attention and cognitive skills among children with ADHD.

**Table 1:** Summary of study traits for virtual reality.

Study	Sample	Focus	Trials and duration	Outcome
Rodrigo-Yanguas et al [17], 2021	N=37 (all subtypes of ADHD^a^); 68% male; age: 12-22 years	To enhance cognitive abilities such as inhibitory control, selective attention, cognitive flexibility, and processing speed	Single session: patients tested each mini-game as long as desired within 1 session	86% reported enjoying playing TSTM^b^ game; 83% expressed interest in continuing to play it; 14% reported dizziness or motion sickness
Stokes et al [19], 2022	N=20; 90% male; age: 10 years (range 8-12 years)	To evaluate whether environmental distractors in the VR^c^ setting would result in lower response rates during behavioral tasks	Single session; 3 blocks of Stroop, Math, and AX-CPT^d^ in a virtual classroom with 200 self-paced trials each; recording responses and eye movement data	Significant decrease in the time spent looking at the whiteboard from the pre- to postdistraction period
Ou et al [20], 2020	N=3; 66% female; age: 9.67 years	To test VR rehabilitation games designed specifically for children with ADHD	36 sessions, 3 times a week for 3 months; 40 minutes per session, divided into four 10-minute game sessions with 5-minute breaks	VR games helped improve attention, impulse control, and defiance in ADHD rehabilitation
Bernardelli et al [18], 2021	The panel involved in this study consisted of 4 experts who all specialized in bioengineering	To develop and test a VR serious game for children with ADHD	Single 30-minute session; task completion times vary by the game level	The study confirmed the feasibility of the VR application for remote performance validation and provided input for possible future improvements
Wiebe et al [21], 2023	N=32; 65.63% female; age: 23.03 years	Feasibility study of VR for assessing attention, impulsivity, and hyperactivity	Only 2 sessions; each of these sessions or blocks had a duration of 24 minutes	Showed the feasibility of the virtual seminar room approach; consistent CPT^e^ performance and no distraction effect; linked inattention and impulsivity

^a^ADHD: attention-deficit/hyperactivity disorder.

^b^TSTM: The Secret Trail of Moon.

^c^VR: virtual reality.

^d^AX-CPT: AX-continuous performance task.

^e^CPT: continuous performance task.

Stokes et al [19] used VR technology integrated with eye tracking to understand attentional distractions in children with ADHD. Participants performed behavioral tasks in a VR classroom while their eye movements were tracked, providing insights into how environmental distractors affect attention. This setup highlighted the feasibility and utility of VR in assessing and understanding attentional processes in ADHD. Similarly, Wiebe et al [21] used a VR seminar room to simulate a classroom environment for assessing attention, impulsivity, and hyperactivity in children with ADHD. The study confirmed the feasibility of such VR setups, demonstrating their potential for detailed assessments of ADHD-related symptoms.

Ou et al [20] employed VR rehabilitation games targeting improvements in attention, impulse inhibition, and oppositional defiance in children with ADHD. Over multiple sessions spanning 3 months, significant positive changes in cognitive and behavioral parameters were observed, demonstrating the effectiveness of VR-based rehabilitation games in managing ADHD symptoms. These studies collectively underscore the effectiveness and feasibility of VR interventions in enhancing cognitive functions and managing ADHD symptoms, highlighting the promise of VR technology as a therapeutic tool for ADHD.

### Motion Sense Devices for ADHD

A summary of motion sense devices for ADHD is presented in Table 2. Gao et al [22] investigated the use of exergaming with motion sense controllers like Xbox Kinect and Wii systems among preschool children. Preschool children engaged in these exergames, which significantly increased their moderate-to-vigorous physical activity (MVPA) levels. This study highlights the potential of exergaming to enhance physical activity engagement in young children.

Benzing and Schmidt [23] focused on children with ADHD, who participated in exergaming sessions aimed at improving executive functions and motor abilities. The intervention led to faster reaction times and improved motor skills, demonstrating the potential benefits of exergaming in enhancing cognitive and motor functions in children with ADHD.

**Table 2:** Summary of study traits for motion sense devices.

Study	Sample	Focus	Trials and duration	Outcome
Gao et al [22], 2020	N=65; 55% female; age: 4.46 years	To assess the effects of exergaming on the physical skills and activity of preschoolers	40 sessions, 5 times a week for 8 weeks; each session: 30 minutes total (20 minutes exergaming and 10 minutes warmup/cooldown)	The exergaming group showed more moderate-to-vigorous physical activity than standard care
Benzing and Schmidt [23], 2019	N=51; 82.4% male; age: 10.43 years	To investigate the effects of cognitively and physically demanding exergaming on executive functions, ADHD^a^ symptoms, and motor abilities	24 sessions, 3 times a week for 8 weeks; each session: 3 hours; participants logged training duration in diaries	The exergaming group had faster reaction times in inhibition and switching tasks and better motor performance after the intervention compared to controls

^a^ADHD: attention-deficit/hyperactivity disorder.

### Exercise-Based Simulators for ADHD

A summary of exercise-based simulators for ADHD is presented in Table 3. Shema-Shiratzky et al [24] combined a treadmill with a VR environment to enhance cognitive and behavioral functions in children with ADHD. Significant improvements were observed in executive function, memory, and gait regularity, alongside reductions in social problems and psychosomatic behavior. This study indicates the comprehensive benefits of combining physical and cognitive training through VR-based interventions. Ko et al [25] explored the use of a ski simulator for VR and non-VR exercises, finding that VR exercises resulted in enhanced exercise capacity and concentration levels, with greater engagement and concentration observed in the VR group compared to the non-VR group. This study underscores the added benefits of VR in exercise-based interventions.

Ji et al [26] evaluated the effects of both exergaming and traditional bicycle exercises in children with ADHD. Both interventions led to improvements in selective attention and self-control, with exergaming showing superior outcomes in cognitive control processes. This study highlights the comparative effectiveness of exergaming over traditional exercises in managing ADHD symptoms, emphasizing the potential of exergaming as a more effective intervention for enhancing cognitive and behavioral functions in children with ADHD.

**Table 3:** Summary of study traits for exercise-based simulators.

Study	Sample	Focus	Trials and duration	Outcome
Shema-Shiratzky et al [24], 2019	N=14; age: 8-12 years	To assess the effects of a VR^a^ training program on executive function and attention abilities in children diagnosed with ADHD^b^	18 sessions, 3 times a week for 6 weeks; each session: 30 minutes to 1 hour	Reduced social problems and psychosomatic behavior; improved executive function, memory skills, and dual-task walking
Ko et al [25], 2020	N=10; mean age 28.50 years (SD 6.11 years)	To evaluate the effects of VR and non-VR exercises on both the exercise capacity and concentration levels when using a ski exergame	Single session; 10 minutes of free stretching, 2 minutes of rest, and 2 sets of 1-minute exercises each, with a 30-minute rest between sets	The results showed that there were significant differences in the VR and non-VR exercises in terms of the range of motion of the ankle and the user’s concentration
Ji et al [26], 2023	N=42; 30% dropout; 87% male; age: 9 years	To investigate the impact of exergaming on attention and self-control in children with ADHD	12 sessions, 3 times a week for 4 weeks; each session: 50 minutes, 60% to 80% of heart rate; 10-minute warmup, 30-minute main exercise, 10-minute cooldown	Both the exergaming group (EXG) and bicycle exercise group had increased selective attention and self-control after exercise; only the EXG showed improved sustained attention and response control

^a^VR: virtual reality.

^b^ADHD: attention-deficit/hyperactivity disorder.

### Areas of Application

The studies reviewed demonstrate the diverse applications of VR and exergaming interventions in managing ADHD symptoms. VR interventions typically focus on cognitive and educational outcomes, employing immersive and interactive environments that engage children dynamically. They are also applied as assessment tools in identifying cognitive traits related to those with ADHD. These interventions have shown significant improvements in attention, memory, and academic skills, as well as high levels of feasibility and acceptance among participants.

In contrast, exergaming interventions primarily aim to enhance physical activity and motor skills through interactive gaming. Studies like those by Gao et al [22] and Benzing and Schmidt [23] highlight the substantial benefits of these interventions in increasing physical activity engagement and improving motor abilities in children with ADHD.

Moreover, studies combining physical and cognitive training, such as those by Shema-Shiratzky et al [24] and Ji et al [26], demonstrate the potential of integrated approaches in providing comprehensive benefits. These interventions not only address cognitive deficits but also enhance physical health and overall well-being, offering a holistic approach to ADHD management.

## Discussion

### Overview

This scoping review aimed to investigate and critically analyze the use, effectiveness, benefits, and potential drawbacks of VR and exercise simulator–based interventions used for individuals diagnosed with ADHD. The core findings indicate that VR-based games and exercise simulators improve attention, inhibitory control, memory, and motor functions, targeting both cognitive and physical deficits in individuals with ADHD. These interventions also significantly increase user motivation and engagement, making them more appealing compared to traditional methods. Participants reported high levels of enjoyment and adherence, which are critical for long-term treatment success.

The review also highlights the importance of tailoring interventions to individual needs, such as adjusting the virtual environments and difficulty levels to ensure their effectiveness across varying symptom severities. The study advances knowledge by demonstrating the dual role of VR and simulators in enhancing cognitive and motor outcomes, advocating for integrated approaches that address the multifaceted nature of ADHD.

In relation to previous literature, the findings are consistent with research on the benefits of physical exercise for ADHD. However, this paper expands the scope by introducing VR and simulator interventions as more engaging and potentially more effective alternatives to conventional exercise and cognitive training programs. Additionally, the review identifies gaps, particularly regarding long-term effects and optimizing user experience to mitigate challenges like motion sickness in VR environments. This comprehensive analysis offers new insights into the role of immersive technology in ADHD treatment while emphasizing the need for further research.

### Impact of VR and Exercise Simulator–Based Interventions

#### Cognitive Function

The selected literature primarily focuses on how VR systems and exercise simulator–based systems can enhance cognitive function in individuals diagnosed with ADHD. For example, VR serious mini-games focused on enhancing cognitive capacity and information processing were implemented in a recent study [17]. This was done through a VR headset using a video game called The Secret Trail of Moon designed through 5 mini-games, with each involving different aspects of cognitive function. For instance, a game called “Enigma” enhances working memory and cognitive flexibility by requiring the user to match elements within a time limit. 

Research studies investigating the use of motion-based controllers in cognitive function typically use VR applications. The study by Ou et al [20] focused on ADHD children, employing a VR intervention and evaluating the outcomes through the Test of Nonverbal Intelligence (TONI-4) and the Wisconsin Card Sorting Test (WCST). The TONI-4 results suggested cognitive advancements after the intervention, while WCST’s outcomes highlighted a mixed response. These findings suggest enhancing cognitive functions using VR warranted exploration. Meanwhile, the study by Benzing and Schmidt [23] employed computer-based tasks, such as the Simon task for inhibition, Flanker task for switching, and N-back task for updating. Most notably, the intervention group had improved reaction times, indicating an enhancement in executive functions. However, no significant changes to the updating abilities were found, leading to suggestions of exploring more sensitive tests for detecting exercise-triggered cognitive changes.

Another study indicated that interventions can effectively target cognitive functions such as working memory and executive function [27]. The study by Shema-Shiratzky et al [24] involving a treadmill with a simulated program evaluated cognitive function using the NeuroTrax computerized neuropsychological battery. This battery includes 5 tasks that encompass various aspects of cognitive function. The tasks include the Stroop test, the Go-No-Go task, verbal and nonverbal memory tasks, and a “Catch game” (measuring set-shifting, adaptation, and planning). The testing for cognitive function was conducted before and after the intervention, as well as during a 6-week follow-up period. The study showed that these interventions had a measured impact on cognitive functions such as working memory and executive function. However, it should be noted that the study did not find significant improvements in the attention score.

#### Attention 

Attention, as articulated by Wu [28] in “We know what attention is,” can be defined as the mental mechanism by which an individual selectively focuses on certain stimuli while disregarding other perceivable information to effectively guide behaviors and responses. This selective process can often be sustained over a period, maintaining a consistent behavioral orientation toward relevant targets; this aspect is termed sustained attention. It involves the ability to persist in cognitive engagement with a single target or task over time, a critical function when continuous monitoring or prolonged concentration is required [28]. Attention can manifest in various forms, from single-mindedly concentrating on one aspect, a concept known as selective attention, to distributing one’s focus across multiple stimuli or tasks, a process referred to as divided attention [28].

This concept forms the basis of a few studies, each offering unique insights into ADHD-related attention processes through various interventions. Bernardelli et al [18] explored selective attention in children with ADHD using a VR application designed to enhance “top-down” and “bottom-up” attention skills, as well as working memory, inhibition, and distributed attention components. The study’s findings indicate that children showed improved ability to filter out distractions and focus on specific tasks within the VR environment, highlighting the potential of VR in enhancing selective attention.

Ji et al [26] focused on sustained attention in children with ADHD through exergaming. This study examined how continuous physical engagement can help children maintain focus over extended periods. The researchers used tools like the Frankfurt Attention Inventory (FAIR) test and the Go-No-Go task, along with monitoring N2 amplitudes associated with conflict monitoring and attentional allocation. The results suggested that sustained physical activity through exergaming significantly improved children’s ability to maintain attention over time.

Stokes et al [19] addressed attentional distraction and selective attention by using VR technology coupled with eye tracking to measure how virtual environments can reduce distractions and enhance selective attention in children with ADHD. Unlike other studies that used VR as an intervention, this study employed it as an assessment tool, demonstrating VR’s potential in evaluating attention difficulties. The study found that children were better able to focus on tasks and reduce distractions when engaging in VR-based activities.

Wiebe et al [21] examined divided attention in adults with ADHD using a multimodal VR-based approach. This feasibility study explored how individuals manage to distribute their focus across multiple stimuli or tasks within a virtual environment, measuring factors such as omission errors, reported distraction levels, and electroencephalography (EEG) metrics like the theta/beta ratio. The findings indicated that VR could effectively help individuals with ADHD improve their ability to manage divided attention.

These studies collectively contribute to understanding the multifaceted nature of attention in individuals with ADHD, providing in-depth insights into how VR and exercise-based simulators can be used to assess and enhance different aspects of attention.

#### Motor Function 

A key theme that emerged from the respective findings is the positive impact of these interventions on cognitive functions, specifically on executive function, in children diagnosed with ADHD. This suggests that interventions using VR and exercise simulator–based systems could potentially address cognitive deficits associated with ADHD. However, only a couple of papers focused on both the cognitive and motor function aspects of these technologies. The impact of a ski exercise–based simulator on exercise capacity and concentration levels was investigated in a study conducted by Ko et al [25]. To assess concentration, the authors used an EEG system and measured motor components such as ankle range of motion and rate of perceived exertion. Benzing and Schmidt [23] used a subset of 6 test items from the German Motor Test to capture various dimensions of motor ability [23]. These dimensions encompass endurance, speed, strength, coordination, and flexibility. Following the intervention, the exergaming group displayed significantly superior overall motor performance compared to the control group. Specifically, improvements in strength and coordination were observed.

This represents a significant void in research as assessing both motor function and cognitive performance is essential for gaining a comprehensive understanding of how VR and exercise-based simulators relate to ADHD. Research has provided unique insights into the importance of assessing motor function in children with ADHD. It underscores significant deficits in motor function in children with ADHD [29,30]. The ambiguity regarding whether motor impairments are inherent to ADHD or triggered by co-occurring developmental coordination disorder has been highlighted, emphasizing the need for more research and the inclusion of motor skills assessment in ADHD care [31]. Children with ADHD were found to have worse dynamic balance performance, another indicator of motor function [32]. In children with ADHD, gross motor function was significantly impaired [33]. Collectively, these studies build a convincing case for exploring motor skills as part of an integrated approach to ADHD diagnosis and treatment. They argue for the necessity to consider these motor impairments when devising treatment plans, to look beyond the evident cognitive aspects of ADHD, and to understand the broader impacts that motor function deficits could have. 

### Perceptions and Experiences

Presenting users in a first-person perspective, VR can create a sense of presence and immersion that enhances engagement and motivation during exercise [34]. VR and exercise simulator–based interventions have shown promise in improving health outcomes and increasing physical activity levels [35]. This was a common theme explored through the literature that was also able to identify similar results with ADHD. The studies by Rodrigo-Yanguas et al [17], Stokes et al [19], and Shema-Shiratzky et al [24] present the recurring theme of interest and enjoyment associated with the use of VR and exercise simulator–based systems. All 3 studies emphasize the role such systems play in enhancing engagement in tasks, fostering better cognitive outcomes, and enhancing the ability to persevere in accomplishing therapy.

Additionally, a study looking at a VR headset focused on the perception of children and adolescents with ADHD [17]. The study considered mental health professionals, such as psychologists and neuropsychologists, and educators, including teachers, pedagogues, counsellors, and others, and found that 40 out of 56 (71%) either provide treatment and education or had provided these services to individuals with ADHD [17]. The perception of participants toward VR was gathered using an ad hoc questionnaire. The questionnaire had specific questions about each game’s mechanics, the VR system’s usability, and the overall experience. The examination of exercise-based simulators and their influence on perception in individuals has not been the focus of any specific studies yet. More direct qualitative responses from participants about their experience and preferences would provide a clearer understanding of their perception toward exercise-based simulators.

### Implications of VR and Exercise Simulator–Based Interventions

#### Positive Changes in Cognitive Domains

The discussed studies provide intriguing implications for the effectiveness of VR and exercise simulator–based interventions for individuals with ADHD. The research studies identified that these interventions bring about positive changes in various cognitive domains. Specifically, improvements have been observed in working memory and executive function, as well as increased levels of concentration. 

#### Efficacy of Exercise-Based Simulators

A study emphasizing selective attention and self-control through exergaming and bicycle exercises demonstrated effectiveness in enhancing cognitive function in children with ADHD [26]. This is evident as both the exergaming group and bicycle exercise group showed significant increases in performance, quality, and continuity values. The mean performance value increased from 190.44 to 269.06 in the exergaming group and from 231.15 to 303.92 in the bicycle exercise group after the intervention, denoting significant improvements (*P*<.001). Similarly, the mean quality value increased in both groups (exergaming group: from 0.90 to 0.95; *P*=.02; bicycle exercise group: from 0.92 to 0.97; *P*=.005). Continuity values also increased significantly in both groups (exergaming group: from 171.33 to 262.44; *P*<.001; bicycle exercise group: from 214.66 to 294.70; *P*<.001). The statistical data implied that the interventions effectively enhanced attention and self-control in children, with slight but greater improvements observed in the exergaming group than in the bicycle exercise group. 

Significant changes in both behavior and cognitive function were observed [24], contributing to existing knowledge in this area. The study used Cohen *d* as a measure of effect size, which is often used to indicate the standardized difference between 2 means [24]. Values are interpreted as follows: 0.2, small effect size; 0.5, medium effect size; and ≥0.8, large effect size. Behavior and social problem measurements had notable effect sizes, with Cohen *d* values of 0.75 and 0.66 for improvements in psychosomatic behavior after training and at follow-up, respectively. Cognitive function showed considerable improvements, especially in executive function and memory. Cohen *d* was reported as 0.63 for the executive function index score and remained >1.01 after a 6-week follow-up for the color trail tests, demonstrating large effect sizes. However, studies on the effects of exercise-based simulators on attention have presented mixed results.

An analysis of sensorimotor rhythm waves associated with concentration revealed that VR exercise resulted in a more pronounced EEG signal compared to non-VR exercise [25]. A more pronounced sensorimotor rhythm wave, often measured with an EEG device, indicates heightened brain activity in the sensorimotor cortex. This area of the brain is associated with processing sensory input and controlling motor functions. A pronounced wave can suggest increased concentration, attention, and engagement in a task. On the other hand, Shema-Shiratzky et al [24] found that VR-based exercise did not significantly improve attention as the attention index scores remained unchanged between testing points. The authors speculated that this could be due to the multifaceted nature of attention, as there are various types of attention, which may not be sensitive enough to reveal changes in different aspects of attention. Some forms of attention may be resistant to VR training effects, and it is plausible that other modalities did not exhibit a response. Furthermore, it has been reported that the attention index score of individuals with ADHD showed no response to either a placebo or methylphenidate, suggesting that some measures of attention may not show changes with various treatments or interventions [36].

### Design and Implementation Considerations

When designing and implementing interventions using VR systems and exercise-based simulators, it is crucial to consider the specific features and requirements that can maximize their effectiveness. Differences in design and implementation across various studies significantly impact the outcomes of these interventions. For instance, a study used a ski exergame sports simulator, while another focused on an exercise-centered treadmill. These variations highlight the importance of carefully considering the type of VR and exercise equipment used when analyzing the effectiveness of these interventions.

#### Case Studies and Examples

Concrete examples of successful serious games and exergaming applications for ADHD provide valuable insights for guiding future designs. For instance, “The Secret Trail of Moon,” a VR game specifically designed for ADHD treatment, effectively enhances cognitive functions such as inhibitory control, selective attention, cognitive flexibility, and processing speed [17]. This aligns with broader findings that engaging game mechanics are crucial for therapeutic success, as they boost user motivation and adherence [37,38]. Additionally, games based on motion-based devices like “Ring Fit Adventure” demonstrate how commercial exergaming can motivate physical activity through fun interactive gameplay, a finding supported by studies highlighting the importance of enjoyment in exergaming for sustained engagement [39]. By analyzing these successful examples, it becomes clear that incorporating immersive and enjoyable elements is essential for maintaining user engagement and achieving desired therapeutic outcomes in ADHD interventions.

#### Exergaming Integration

The integration of exergaming into ADHD interventions is supported by a growing body of literature emphasizing the benefits of physical activity for cognitive and motor development [26,40,41]. Effective integration involves the use of hardware, such as motion sensors and VR headsets, to create interactive and engaging experiences. Studies show that motion sense controllers like Xbox Kinect or Nintendo Switch significantly increase MVPA levels, particularly in young populations [22]. For ADHD interventions, game mechanics should blend physical activity with cognitive challenges. Tasks like obstacle courses requiring quick decision-making or following complex instructions while physically active can enhance both motor skills and executive functions [23]. By incorporating these elements, interventions align with guidelines on promoting physical activity as a core component of ADHD treatment [9].

#### Accessibility and Inclusivity

Ensuring accessibility and inclusivity is a critical aspect of designing effective VR and exergaming interventions, as highlighted in existing guidelines on technology-assisted therapies [42]. Adjustability in difficulty levels and customizable controls are recommended to cater to users with diverse needs and preferences [21]. Studies have emphasized that settings allowing users to modify task intensity and complexity ensure that the intervention remains appropriately challenging for individuals with varying skill levels [43]. Moreover, diverse representations in games, including characters and scenarios reflecting different cultures and backgrounds, are crucial for increasing engagement across broad user groups [44]. Accessibility should also consider physical adaptations, such as seated play options or alternative control schemes, ensuring that users with physical disabilities can benefit from the interventions. These considerations reflect best practices in inclusive game design, promoting broader access and greater adherence to treatment programs.

### Strengths and Weaknesses of Exercise-Based Simulators and VR

Exercise simulators offer a robust solution for managing ADHD by integrating physical activity with cognitive training. As detailed in Table 4, the design features identified through various studies, such as immersive VR environments, motion sense controllers, and customizable settings, can be effectively applied to the exercise simulator. By combining cognitive training elements with realistic and engaging VR experiences, the sailing simulator can provide a comprehensive therapeutic tool for individuals with ADHD. Emphasizing user safety, engagement, and adaptability ensures that the intervention is both effective and accessible, ultimately improving cognitive and behavioral outcomes for users.

While the studies revolving around exercise-based simulators had not focused on the perception of the participants, all of them did mention a few positive points related to the experience. For example, 1 study identified that the game-like properties of the exercise simulator potentially made the training regimen more engaging, likely promoting participants’ adherence to the program and being effectively used by individuals who generally do not prefer traditional exercise but enjoy gaming [24]. The 3 studies found no adverse effects linked to the use of exercise-based simulators. However, a potential limitation that was identified is the lack of variability in the types of games or exercises that can be played on these devices. Since most simulators are designed around specific sports or activities, there may be limited options for different types of exercises or games to be performed on these devices.

Motion-based devices coupled with serious games present a compelling avenue for interventions, particularly for individuals with ADHD. The consensus points toward the engaging nature of these tools, using movement and interactivity to capture attention and promote short-term participation, a known challenge in traditional ADHD interventions. This form of technology can potentially enhance motivation and short-term adherence to a therapeutic program due to its active style. While research is ongoing, preliminary findings suggest that motion-based serious games can yield improvements in areas like attention, executive function, and motor coordination, all of which are commonly affected in ADHD. However, a further investigation is needed to solidify these findings and determine the long-term efficacy. Despite the promising outlook, considerations regarding the inherent structure of games, with defined levels and goals, could lead to a plateau in learning and engagement once those targets are achieved. This inherent limitation might result in diminished motivation over time, particularly for individuals with ADHD who thrive on novelty and challenge. Alternatively, exercise-based simulators, grounded in real-life sports and activities, offer a more open-ended experience. The pursuit of mastery in activities like cycling or sailing, with their nuanced techniques and physical demands, presents a continuous challenge that surpasses the limited nature of game-based scenarios. The open-ended nature of exercise-based simulators, which allows for continuous skill development and application in real-world scenarios, aligns well with the need for sustained engagement in ADHD interventions. This approach may lead to greater long-term adherence and more impactful therapeutic outcomes compared to more constrained game-based scenarios.

In contrast, VR systems offer a wide range of environments and experiences, providing a unique platform for various interventions. Studies have highlighted the effectiveness of VR-based interventions in managing ADHD. For example, Rodrigo-Yanguas et al [17] reported high levels of user satisfaction, with 86% of participants enjoying the game, 83% wanting to replay it, 78% finding it easy to understand, and all participants feeling good after playing. This suggests that children with ADHD prefer VR-based cognitive tests over traditional ones, which has also been noted by Fang et al [45].

The VR-based “virtual seminar room” has proven to be effective in ADHD assessment by offering a standardized yet realistic setting. Wiebe et al [21] found that this environment enhances ecological validity and provides insights into participants’ distractibility and impulsiveness, potentially improving ADHD evaluations. Such positive engagement in VR experiences may lead to increased task endurance among children with ADHD.

Bernardelli et al [18] conducted a comprehensive analysis of VR interventions for children with ADHD, emphasizing their dual benefits for cognitive training and physical activity. Their findings indicated improvements in both attention and motor skills, underscoring the versatility of VR systems in ADHD treatment. The study also highlighted the need for personalized VR settings to minimize discomfort and maximize engagement, reinforcing the importance of tailored approaches in VR interventions.

Despite the numerous benefits, VR interventions are not without drawbacks. Approximately 14% of users reported discomfort, and motion sickness was a significant concern [17]. Addressing these issues is crucial to optimize the effectiveness of VR-based therapies.

**Table 4:** Summary of the strengths and weaknesses found through exercise simulators and virtual reality.

Study	Technology used	Strengths	Weaknesses
Rodrigo-Yanguas et al [17], 2021	VR^a^ headset, console (PlayStation 4), and serious game: The Secret Trail of Moon (TSTM)	High interest and enjoyment: 83% of children expressed interest in playing. Most found the instructions easy to understand and the games easy to play	14% reported discomfort or motion sickness from VR glasses, with dizziness being a potential side effect
Stokes et al [19], 2022	VR headset (HTC Vive), PC, eye tracking (SensoMotoric Instruments), and 3D-simulated classroom	Children with ADHD^b^ found VR cognitive testing more enjoyable than traditional tests, potentially increasing persistence.	VR classrooms have shown worse behavioral performance in other studies
Ko et al [25], 2020	Ski exergame (Ski Fit 360)	Enhancement of exercise and concentration: VR increased exercise capacity, with wider ankle motion and higher SensoMotoric Rhythm (SMR) wave activity indicating improved concentration	The ski motion platform made it difficult for users to maintain balance when moving side to side
Ji et al [26], 2023	Exergame group: ExerHeart devices (D&J Humancare App Alchemist’s Treasure); Nonexergame group: Stationary bike (Fit Elite-Whole body exerciser 100)	Improved attention and control: exergaming improved selective attention and self-control, with N2 amplitude changes indicating better cognitive control	Only 1 game could be played on the simulator, limiting variety
Shema-Shiratzky et al [24], 2019	Exergame: the system utilized projects a computer-generated simulation in front of a treadmill	Gait and cognitive improvements: VR improved gait regularity, executive function, and memory, and reduced social and psychosomatic issues in children with ADHD	No negatives or limitations were mentioned regarding the simulator setup
Benzing and Schmidt [23], 2019	Motion sense controller, console (Xbox Kinect), and serious game: Shape Up	Improved reaction times and motor skills: exergaming led to faster reaction times and better motor abilities; participants rated enjoyment highly (3.62/4)	High dropout rate; no significant improvements in some tasks like updating; general health benefits were seen, but not specific ADHD symptom improvements
Ou et al [20], 2020	VR headset (HTC Vive) and PC game: “Fishing Master, Fruit Train and Ocean Manager”	Cognitive and behavioral enhancements: participants showed cognitive improvements and reductions in ADHD symptoms, such as inattention and hyperactivity	Participants lost interest after mastering the games, and frequent breaks were needed to prevent discomfort from the VR headgear
Bernardelli et al [18], 2021	VR headset (Oculus Quest) and PC serious game: ADAD	User-friendly VR application: VR reduced impulsivity and improved attention, with a customizable intervention for individual needs	Need for automatic level selection to reduce errors; remote implementation complexities affected operations
Gao et al [22], 2020	Motion sense controller, console (Wii or Xbox Kinect) games: Just Dance for Kids (both consoles), and Wii Nickelodeon Fit	Engagement in physical activity: participants found the intervention engaging, spending 40% of school time in moderate-to-vigorous physical activity	No negatives or limitations were mentioned regarding the intervention setup
Wiebe et al [21], 2023	A virtual seminar room (VSR) was created using Unity 3D and featured a VR headset (HTC Vive) and PC, replicating a real-world seminar room with typical furniture, a front canvas, and virtual classmates	Feasibility of the VSR: the VSR was well-accepted, with successful behavioral and neurophysiological data recording and low cybersickness reports	Head movements and strain from the VR headset caused artifacts that interfered with electroencephalography signals; cybersickness was still reported

^a^VR: virtual reality.

^b^ADHD: attention-deficit/hyperactivity disorder.

### Limitations

Despite the promising results of integrating VR and exercise-based simulators for managing ADHD, some limitations must be acknowledged.

#### Methodological Limitations

Despite employing a structured search across multiple databases, this review may be susceptible to publication bias, potentially favoring studies that report positive or significant findings. As a result, there is a risk of overestimating the effectiveness of VR and exercise-based interventions, as studies with neutral or negative outcomes might be underrepresented. Additionally, the search strategy, while comprehensive, focused on specific terms such as “virtual reality,” “exercise simulator,” “exergaming,” and “serious games.” This narrow focus may have unintentionally excluded relevant studies that use different terminology or related technologies. Furthermore, the decision to limit the search to articles published between 2019 and 2023 may have omitted earlier foundational research, limiting the understanding of the field’s broader development and historical context.

Another limitation is language and population bias. By including only English-language publications, the review may have excluded valuable research conducted in other languages, which could provide additional insights. Moreover, the review’s focus on human participants diagnosed with ADHD may have inadvertently excluded research on other populations, such as those with cognitive impairments or neurodevelopmental disorders that share overlapping symptoms, potentially limiting the scope of findings that could otherwise be applicable to ADHD management.

#### Limitations Within the Included Studies

Many of the included studies featured relatively small sample sizes, which limits the generalizability of their findings to broader ADHD populations. Larger and more diverse samples are needed to validate these results and ensure their applicability to a wider demographic. Additionally, most studies lacked long-term follow-up data, making it difficult to assess whether the short-term benefits of VR and exercise-based interventions are sustained over time. Longitudinal studies are essential to evaluate the durability and potential long-term effects of these interventions. Furthermore, several of the studies lacked rigorous design frameworks, such as randomized controlled trials, which are critical for establishing clear causal relationships between the interventions and the observed improvements in ADHD symptoms. This absence of methodological rigor weakens the strength of the conclusions drawn from the available evidence.

These limitations underscore the need for cautious interpretation of the review’s findings. While the review offers valuable insights, it should be seen as a foundation for further investigation. Future research should prioritize larger and more diverse samples, long-term follow-up, and rigorous study designs, including randomized controlled trials, to assess the potential of VR and exercise-based simulators more fully for managing ADHD.

### Conclusion

VR and exercise-based interventions show promise as potentially effective approaches for people with ADHD. Studies suggest that individuals with ADHD find VR cognitive testing and experiences more pleasurable compared to traditional tests, which may result in increased engagement and persistence in tasks. The use of VR in ADHD interventions offers a wide range of virtual environments and activities that can be tailored to individual needs, providing a more versatile and engaging experience for participants. Both forms of technology have also shown improvements in a variety of cognitive functions. However, it is crucial to recognize the potential drawbacks of VR interventions, including certain users experiencing discomfort and dizziness. These negative reactions can impact adherence to the program and add additional cognitive load during tasks, potentially compromising the effectiveness of the intervention. Previous research conducted on exercise-based simulators did not consider the perspectives of individuals with ADHD toward this type of intervention. This implies that there is a gap in understanding how people diagnosed with ADHD perceive and respond to exercise-based simulators. It is important to address such misunderstandings so that people with ADHD can benefit from the opportunities provided by VR and exercise-based interventions to support the challenges they face with ADHD, which will enable them to enjoy a higher quality of life and a normal life.

## Appendix

Multimedia Appendix 1 PRISMA (Preferred Reporting Items for Systematic Reviews and Meta-Analyses) checklist.

## Abbreviations

ADHD: attention-deficit/hyperactivity disorder
EEG: electroencephalography
MVPA: moderate-to-vigorous physical activity
PRISMA-ScR: Preferred Reporting Items for Systematic Reviews and Meta-Analyses Extension for Scoping Reviews
TONI: Test of Nonverbal Intelligence
VR: virtual reality
WCST: Wisconsin Card Sorting Test
